# Autoantibodies in Spondyloarthritis, Focusing on Anti-CD74 Antibodies

**DOI:** 10.3389/fimmu.2019.00005

**Published:** 2019-01-22

**Authors:** Yuan Liu, Xining Liao, Guixiu Shi

**Affiliations:** ^1^Department of Rheumatology and Clinical Immunology, The First Affiliated Hospital of Xiamen University, Xiamen, China; ^2^Medical College, Xiamen University, Xiamen, China

**Keywords:** spondyloarthritis, autoantibodies, diagnosis, anti-CD74 autoantibody, Chinese patients

## Abstract

Spondyloarthritis (SpA) is an inflammatory rheumatic disease with diverse clinical presentation. The diagnosis of SpA remains a big challenge in daily clinical practice because of the limitation in specific biomarkers of SpA, more biomarkers are still needed for SpA diagnosis and disease activity monitoring. In the past, SpA was considered predominantly as auto-inflammatory disease vs. autoimmune disease. However, in recent years several researches demonstrated a broad autoantibody response in SpA patients. Study also indicated that mice lack of ZAP70 in T cell develop SpA featured inflammation. These studies indicated the autoimmune features of SpA and gave rise to the potential use of autoantibody in SpA management. In this article, we reviewed recent reports of autoantibodies associated with SpA patients, revealing the autoimmune features of SpA, suggesting the hypothesis that SpA was also an autoimmune disease, studies about the autoimmune features might provide more insights in the pathogenesis of SpA. In addition, as there are two opposite conclusions in the role of anti-CD74 autoantibody in the diagnosis of SpA, we also gave our own data on the diagnostic value of anti-CD74 in Chinese SpA patients. Though our data indicated that anti-CD74 might not be a good biomarker for SpA diagnosis in Asian people, CD74 was still a good molecule target in the research of SpA pathogenesis.

## Introduction

Spondyloarthritis (SpA) is an inflammatory rheumatic disease with diverse clinical presentation, including psoriatic arthritis, reactive arthritis, arthritis related to inflammatory bowel disease, a subgroup of juvenile idiopathic arthritis, and ankylosing spondylitis (AS) (1). The diagnosis of SpA remains a big challenge in daily clinical practice because of the limitation in specific biomarkers of SpA. Currently, HLA-B27 is the most widely used genetic biomarker in SpA diagnosis and CRP seems to be the best biomarker in evaluating disease activity of SpA (2). However, HLA-B27 can also be detected in healthy person and have no association with clinical presentation (3). Increased CRP lacks specificity in SpA because it can be detected in a large variety of inflammatory situations and it is found to be significant lower when new syndesmophytes development (4). Studies in recent years highly recommend the use of magnetic resonance imaging (MRI) in early diagnosis because its high sensitivity in detecting active sacroiliitis (5). However, MRI inflammatory abnormalities can also be detected in other diseases such as infection and it is costly in daily clinical practice for monitoring SpA. More biomarkers are still needed for SPA diagnosis and disease activity monitoring.

In the past, most immunologists believed the predominance of innate vs. adaptive immune responses in SpA, indicating a prominent autoinflammatory component in pathogenesis (6). Since SpA was considered as an inflammatory disease, autoantibodies were rarely used in diagnosis of SpA. However, several studies in recent years detected some autoantibodies in serum of SpA patients and also demonstrated a broad autoantibody response in these patients. Furthermore, as human immune response is a network, innate and adaptive immune responses promote and connect with each other and often accompanied by secondary T-cell and B-cell activation, especially several studies indicated that Th17 cells might be involved in the pathogenesis of SpA (7, 8). In addition, study also showed that mice lack of ZAP70 in T cell developed SpA featured inflammation (9). These studies revealed the autoimmune features of SpA and give rise to the hypothesis that SpA was also an autoimmune disease and the potential use of autoantibody in SpA management (10).

Presence of autoantibody is a key characteristic of autoimmune disease. Autoantibody is one of the most common biomarkers used in diagnosis and disease activity evaluating of autoimmune disease, it shows good sensitivity because it can sometimes be detected in serum years before the abnormal state of antigen been detected. In this article, we reviewed recent reports of autoantibodies associated with SpA patients to further reveal the autoimmune features of SpA and potential autoantigens. In addition, as there are two opposite conclusions in the role of anti-CD74 in the diagnosis of SpA, we also studied the presence of anti-CD74 in Chinese SpA patients.

## Autoantibodies in SpA Patients

### Anti-beta2 Microglobulin Autoantibodies

Beta 2 microglobulin, also known as B2M, it is the light chain of the human lymphocyte antigen (HLA) and a component of major histocompatibility complex (MHC) class I molecules on all nucleated cells (11, 12). In normal adults, there are small amounts of beta 2 microglobulin in serum, and ordinarily remained at a relatively stable level. However, in exceptional circumstances, the level of beta 2 microglobulin in serum would be change, like renal failure, after a trauma and tumor. Some investigators have found that the expression of beta 2 microglobulin elevated in some immune diseases, such as systemic lupus erythematosus (SLE) (11, 13), rheumatoid arthritis (RA) and Sjögren's syndrome (14–16), and anti-beta2 microglobulin was also detected in SLE patients (17–19). In order to explored the presence of anti-beta2 microglobulin in other common rheumatic diseases, Russell Curry et al. detected the expression of anti-beta2 microglobulin in AS, SLE, RA patients, and healthy controls. They showed that IgG antibody to beta 2 microglobulin was found in 68% of 22 patients with AS, although the presence of IgG anti-beta2 microglobulin was 71% in 35 SLE patients, the level of anti-beta2 microglobulin in AS patients was highest in all groups (20). Regrettably, we did not find any further investigations of this antibody in AS.

### Anti-mutated Citrullinated Vimentin Autoantibodies (Anti-MCV)

Anti-citrullinated protein/peptide autoantibodies (ACPAs) are widely used in diagnosis of rheumatoid arthritis (RA), but their relationship with SpA is still unclear. Some recent studies used genetically modified, mutated citrullinated vimentin (MCV) as auto-antigen to detected antibodies to citrullinated vimentin (CV). One research indicated that the serum anti-MCV level was higher in patients with AS in comparison to healthy controls, and the prevalence of anti-MCV in AS patients was 37% compared with 0% in healthy controls. In this study, there was no significant difference in the expression of this autoantibody in different types of spondylitis. Furthermore, anti-MCV positivity was correlated with erythrocyte sedimentation rate (ESR) in AS patients, but no correlation with age, disease duration, C reactive protein (CRP), HLA-B27 status, smoking habits, pain intensity (VAS), Bath Ankylosing Spondylitis Disease Activity Index (BASDAI), Bath Ankylosing Spondylitis Functional Index (BASFI) or Bath Ankylosing Spondylitis Metroloty Index (BASMI)(21). Another research compared the diagnostic value of anti-MCV in differentiating early inflammatory arthritis, they found 13.9% patients in AS can be detected anti-MCV in their serum, while 15.2% in psoriatic arthritis and 62% in rheumatoid arthritis patients (22).

### Anti-heat Shock Protein 65 Autoantibodies (Anti-HSP65)

Heat shock proteins (HSPs) are highly conserved proteins with protective functions in situations of cellular stress. HSP65 was one of HSPs named according to its molecular weight. Autoantibodies to HSPs had been found in several kinds of diseases including cancer and inflammatory disease. Recently, higher level of anti-HSP65 in patients with AS than in healthy controls was shown in a research, but the number of patients included in this study was relatively small, they only compared 43 AS patients with 11 patients with low back pain but no AS (21). On the contrary, another study indicated that although anti-hsp65 was elevated in 19/59 patients (32%), the level of elevation was not significant. At the same time, they also detected the expression of anti-hsp65 in RA, in contrast, significantly elevated IgA anti-HSP65 was observed in RA (23).

### Anti-14-3-3 eta Autoantibodies (Anti-14-3-3η)

Traditionally, 14-3-3η (eta) is regarded as an intracellular protein. Like other intracellular protein, such as HSPs, upon some stressful conditions, 14-3-3η (eta) can be released into the extracellular compartments, and elicits a specific auto-antibody response (24–26). It has been reported that anti-14-3-3η (eta) has specificity and sensitivity in patients with early RA and that it may be used for the diagnosis of early RA, at the same time, in the control groups, high level of anti-14-3-3η (eta) was observed in patients with AS (27). Subsequently, Maksymowych et al. further detected the expression of anti-14-3-3η (eta) in patients with AS and assessed the relationship between anti-14-3-3η (eta) and sacroiliac joints (SIJ) inflammation. They found that anti-14-3-3η (eta) was significantly increased in sera of AS patients and correlated with SIJ inflammation, C-reactive protein (CRP), baseline modified stoke ankylosing spondylitis spine score (mSASSS), and change in mSASSS in AS patients (28).

### Anti–protein Phosphatase Magnesium-Dependent 1A Autoantibodies (Anti-PPM1A)

Differentiation of osteoblasts leads to syndesmophytes formation is a key feature of AS, this process requires the participation of some signaling pathways, such as bone morphogenetic proteins (BMPs), Wingless proteins (Wnt), and so on (29). Protein Phosphatase Magnesium-Dependent 1A (PPM1A) is a serine/threonine (Ser/Thr) protein phosphatase known as an inhibitor of tumor growth factor (TGF)-β signaling pathway, participates in bone formation by regulating BMP and Wnt signaling pathway (30, 31). High-density protein microarrays showed that higher auto-reactivity against PPM1A was detected in AS patients than in other autoimmune diseases. Then, in Korean patients, compared with RA patients (*n* = 20) and healthy controls (*n* = 30), significantly higher levels of anti-PPM1A were observed in treatment-naïve patients with AS (*n* = 45). Furthermore, AS patients with grade 3 or 4 radiographic sacroiliitis, the levels of anti-PPM1A were also higher than in those with grade 2 radiographic sacroiliitis. After treatment with a TNF inhibitor, the serum levels of anti-PPM1A were significantly decreased in patients with AS, and correlated positively with BASDAI score. These results indicated that anti-PPM1A autoantibodies might not only serve as a diagnostic biomarker, but also associated with severity of sacroiliitis, and might be used as a predictor of response to anti-TNF therapy in AS patients. In addition, they also found that the expression of PPM1A was increased in AS patients, indicated that PPM1A might also be involved in pathogenesis of AS (31).

### Anti-sclerostin Autoantibodies (Anti-SOST)

As mentioned earlier, bone formation plays an important role in the pathogenesis of AS (32), and as the expression product of the sclerostin gene (SOST), sclerostin is an inhibitor of bone formation and plays a crucial role in the formation of bone (33). One study had shown that in the comparison of AS, RA, osteoarthritis (OA) and healthy controls, lower sclerostin level was observed in OA and AS patients, especially in AS patients (34). Meanwhile, another study had indicated that the overexpression of anti-SOST would lead to lower level of SOST (35). Based on these research results, Michele Maria Luchetti et al. further studied the significance of anti-SOST-IgG in the diagnosis of SpA, they focused their attention on spondyloarthritis-associated inflammatory bowel diseases (SpA/IBD), one of the group of SpA. They found that the level of anti-SOST-IgG was significantly higher in axial spondyloarthritis-associated inflammatory bowel diseases (axSpA/IBD) patients compared with peripheral SpA/IBD (per-SpA/IBD) and IBD patients (43.29 ± 13.74, 21.33 ± 11.33, 27.27 ± 11.77 IU/ml, respectively), and they also found anti-SOST-IgG serum levels were inversely associated with the duration of articular symptoms (36). They indicated that anti-SOST-IgG might be used as a potential biomarker in axial SpA in patients with IBD, in addition, the presence of anti-SOST-IgG and reduction of SOST might be used as a new target in the study of SPA pathogenesis.

### Antibodies Against Microbial Targets

Several antibodies against microbial targets have been detected in serum of inflammatory bowel disease (IBD) patients, suggesting loss of tolerance to a subset of commensal microorganisms (37). These include anti-Saccharomyces cerevesiae antibodies (ASCA) directed against a cell wall polysaccharide of the yeast, anti-neutrophil cytoplasmic antibodies (pANCA), anti-I2 (associated with anti-Pseudomonas activity), anti-Eschericia coli outer membrane porin C (anti-OmpC), and anti-flagellin (anti-CBir1). As AS patients share many similarities with IBD patients, those antibodies have also been detected in serum of AS patients. Increased prevalence of ASCA, ANCA, and anti-CBir1 were found in AS patients by some studies (38–40), while other studies showed no significant difference in positivity rates of those antibodies in AS patients (41, 42). Clinical significance of those antibodies in SPA patients still needed to be evaluated.

### Anti-CD74 Autoantibodies

#### Anti-CD74 Autoantibody in European SpA Patients

The human CD74, also known as human lymphocyte antigen (HLA) class II gamma chain or invariant chain, is considered as part of formation and transport of major histocompatibility complex class II (MHC II) molecules (43). There are two different domains in the extracellular part of CD74, class II-associated invariant chain peptide (CLIP) was one of them. The main function of CLIP is to prevent the binding of premature peptide to newly assembled class II HLA molecules (44–46). Some researchers have found that the interaction between anti-CLIP antibodies and CD74 may produce proinflammatory cytokines, such as tumor necrosis factor α (TNFα) (47). Recently, some German researchers screened new autoantigens by using protein array technology based on cDNA of fetal brain tissue. A total of 59 sera were studied, including different inflammatory and rheumatic diseases (*n* = 55, 5 radiographic axial SpA) and 4 healthy controls. IgG antibodies against CD74 were detected in 4/5 radiographic axial SpA patients, while in 54 controls, only 1 of them was detected IgG antibodies against CD74. Since there has been little research on the role of another extracellular part of CD74, thyroglobulin type-1 in CD74, the researchers focused their attention on CLIP. So, they detected the serum level of anti-CD74 and anti-CLIP in patients by ELISA, the results showed anti-CD74 IgG protein in 23/41 (56%) patients with radiographic axial SpA, compared with 5/100 (5%) in blood donors, meanwhile, anti-CLIP IgG was found to present in sera of 67% SpA patients and was even more frequent in patients with a short duration, but had no association with disease activity, indicating it might be used as a biomarker in the early diagnosis of SpA. This study included 216 SpA patients from Hannover, Berlin and Vienna (48). Another European study also came to similar results (49). These studies provided preliminary evidence that anti-CD74 antibody might be a promising biomarker in SpA diagnosis.

However, a study indicated that anti-CD74 IgG and IgA autoantibodies have no diagnostic value in early axial spondyloarthritis (50). In this study, compared with patients with chronic back pain (CBP), the level of serum anti-CD74 IgG autoantibody did not elevate in early axSpA patients, and the difference in prevalence of anti-CD74 IgG autoantibody in axSpA and CBP patients was not significant (46.4, 47.9%, respectively). On the other hand, although level of anti-CD74 IgA was higher in patients with early axSpA, this elevation was not sufficiently specific to yield significant diagnostic value in patients under 45 years old presenting with early back pain.

#### The Presence of Anti-CD74 Autoantibody in Chinese SpA Patients

**Figure 1:**
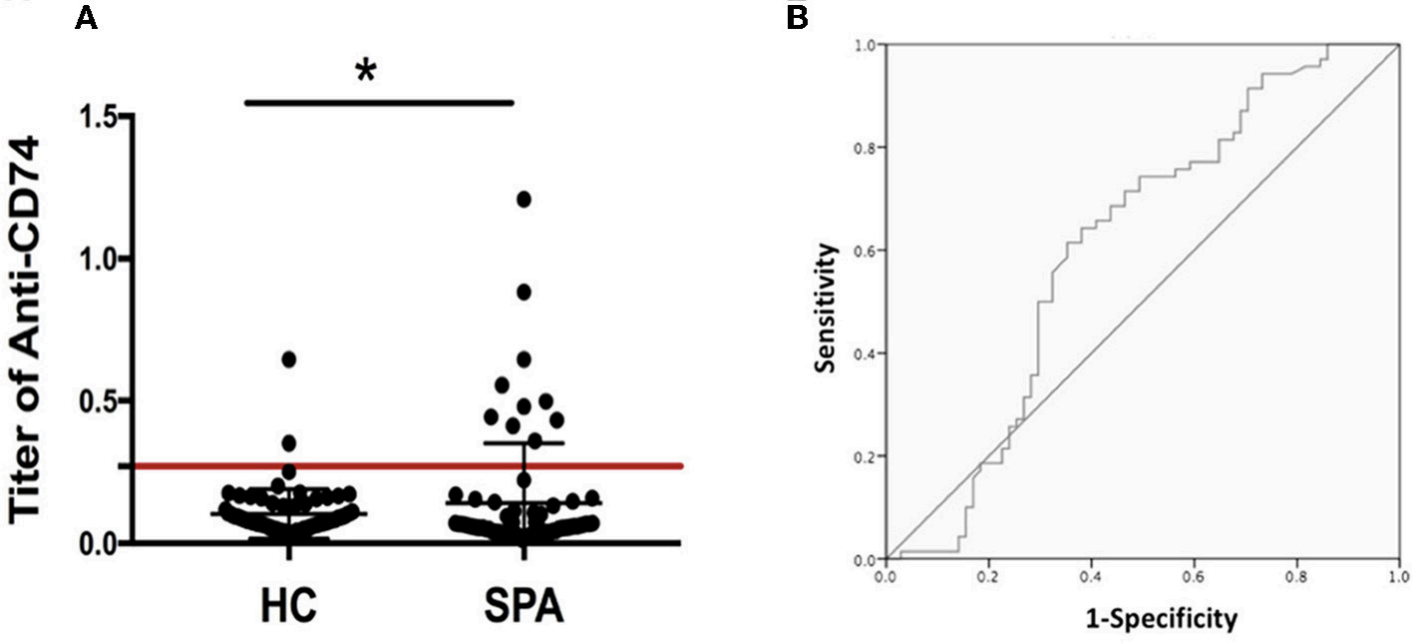
**(A)** Titer of anti-CD74 autoantibody by ELISA. Titer of anti-CD74 in SpA serum was higher than that in healthy controls, but the prevalence of anti-CD74 was not as high as the results from the Europe cohort. The cutoff value line for positive samples is indicated in the figure. **(B)** Receiver operating characteristic curves (shown as area under the curve [AUC]) demonstrated the sensitivity and specificity of anti-CD74 Ab in diagnosis of SpA. AUC = 0.608, with 95% confidence intervals of 0.513–0.704. *p < 0.05.

**Figure 2:**
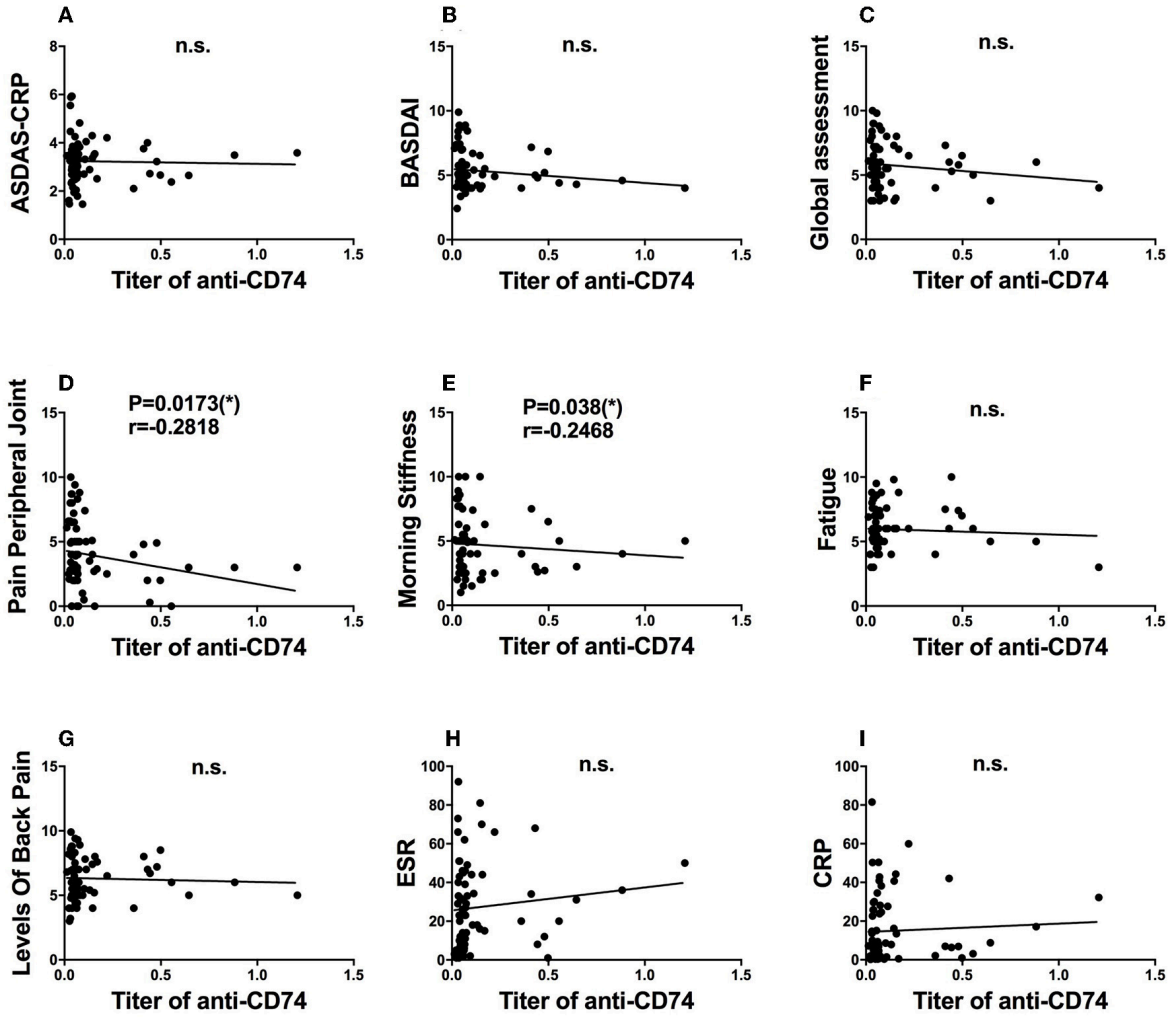
Clinical significance of anit-CD74 autoantibody in SpA patients. Association of titer of anit-CD74 and ASDAS-CRP **(A)**; BASDAI **(B)**; patient global assessment **(C)**; pain peripheral joint **(D)**; morning stiffness **(E)**; fatigue **(F)**; levels of back pain **(G)**; erythrocyte sedimentation rate (ESR) **(H)** and C reactive protein (CRP) **(I)** were determined by using spearman's correlation test, no significant correlation was found between titer of anti-CD74 and these disease activity index, except pain peripheral joint morning stiffness.

As the role of anti-CD74 in the diagnosis of SpA is still controversial, we further investigated presence of anti-CD74 in sera of Chinese SpA patients by ELISA. A total of 141 sera obtained from the serum bank of the First Affiliated Hospital of Xiamen University, including 71 patients with SpA and 70 healthy controls (HC). All the sera had been stored for less than 1 year at −80°C before they were studied. All patients were diagnosed by the rheumatologist, and fulfilled the modified New York (mNY) criteria for AS. The medical ethic committee of The First Affiliated Hospital of Xiamen University has approved this study. We found that although the mean titer of anti-CD74 in SpA patients was higher than healthy controls, the prevalence of anti-CD74 was not as high as the results from the Europe cohort (Figure 1A). We also used the receiver operating characteristic curves (ROC) to demonstrate the sensitivity and specificity of anti-CD74 in diagnosis of SpA (Figure 1B). The ROC curve revealed not very strong performance characteristics of anti-CD74 in Chinese SpA patients, with AUC values of 0.608, and 95% confidence intervals of 0.513–0.704. By using the mean titer of HC plus two SDs as the cut-off value to determine the sera samples with positive anti-CD74, the prevalence of anti-CD74 in Chinese SpA patients was 14.1%, and 2.9% in healthy controls (Table 1). We also analyzed the correlation of titer of anti-CD74 and Ankylosing Spondylitis Disease Activity Score-C Reactive Protein (ASDAS-CRP), Bath Ankylosing Spondylitis Disease Activity Index (BASDAI), patient global assessment, swelling/pain peripheral joint, morning stiffness, fatigue, levels of back pain, erythrocyte sedimentation rate (ESR) and C reactive protein (CRP). As shown in Figure 2, no significant correlation was found between titer of anti-CD74 and these disease activity indexes, except swelling/pain peripheral joint, morning stiffness (Figure 2).

**Table 1:** Frequency of autoantibody against CD74 in human sera by ELISA.

	**Number**	**Anti-CD74 (+)**	**Frequency (%)**
HC	70	2	2.9
SPA	71	10	14.1^*^

*HC, healthy control; SPA, spondyloarthritis*.

* *P < 0.05*.

We used Graphpad prism 7.0 for statistical analysis. Comparisons between groups were evaluated using the Mann–Whitney *U* test. For correlations we used the Spearman test. Statistical analysis was performed in SPSS 20.0 software, and *P* < 0.05 was considered statistically significant.

#### Possible Reasons for Difference in Diagnostic Value of Anti-CD74 Among Studies and Possible Role of CD74 on the Pathogenesis of SpA

Our study showed prevalence of anti-CD74 was not as high as the results from the Europe cohort. This might result from difference in study population, indicating that anti-CD74 might not be a good biomarker for SpA diagnosis in Chinese patients. Also, as discussed in the study of anti-CD74, soluble CD74 was presented in fresh sera that may interfere the binding of autoantibody to CD74 with CD74 on ELISA plate, the sera used in the study of anti-CD74 were frozen for years, in which soluble CD74 appeared to be slowly degraded after years (48). The sera in our study were kept froze less than 1 year, this might be one of the factors contribute to low prevalence of anti-CD74 in our study.

CD74 has been proved to be one of the key regulators in inflammation by many studies. It is expressed on several types of immune cells such as dendritic cells, macrophages and B cells, performed activities in antigen presentation, B cell differentiation, motility of dendritic cells and thymic selection (51), which are all important in pathogenesis of inflammation. Activation of CD74 initiates signaling cascade which leading to NF-κB activation (52), which are important in production of proinflammatory cytokines. Many studies have demonstrated role of CD74 in several kinds of inflammatory or autoimmune diseases such as atherosclerosis, lupus, and diabetes mellitus (51). It can be speculated that CD74 might be involved in pathogenesis of SpA from role of CD74 in inflammation. Autoantibodies to CD74 might be involved in pathogenesis of SpA by activating CD74 and leading to production of proinflammatory cytokines. Though our studies showed that anti-CD74 might not be a good diagnostic biomarker for SpA patients in Asian, CD74 is still a good molecule to be studied in SpA pathogenesis mechanism and treatment.

## Conclusion

SpA was long considered as an autoinflammatory disease and researches about biomarkers of SpA mainly focused on innate immunity components. As presence of autoantibodies in serum of SpA patients was found by studies, the hypothesis that SpA was also an autoimmune disease has been proved gradually, the involvements of adaptive immune system in the pathogenesis will attract more attention to rheumatologists as well as clinical immunologists. In the future, autoantibodies in SpA patients need to be detected in different populations and large number of SpA patients. As the involvements of adaptive immune system in the pathogenesis being revealed, autoantibodies and cellular immune components may become important biomarker as well as new therapeutic targets, which may greatly improve the diagnosis and management of SpA patients.

## Author Contributions

All the authors were involved in the design of the study and in writing the manuscript. YL and XL participated in the study design, literature review, collected serum samples, and performed statistical analysis and presentation of the results and participated in the drafting and review of the manuscript. GS participated in the study design, and participated in the drafting and review of the manuscript.

### Conflict of Interest Statement

The authors declare that the research was conducted in the absence of any commercial or financial relationships that could be construed as a potential conflict of interest.
